# Patterns of Rift Valley fever virus seropositivity in domestic ruminants in central South Africa four years after a large outbreak

**DOI:** 10.1038/s41598-020-62453-6

**Published:** 2020-03-26

**Authors:** Yusuf B. Ngoshe, Alida Avenant, Melinda K. Rostal, William B. Karesh, Janusz T. Paweska, Whitney Bagge, Petrus Jansen van Vuren, Alan Kemp, Claudia Cordel, Veerle Msimang, Peter N. Thompson

**Affiliations:** 1 Epidemiology Section, Department of Production Animal Studies, Faculty of Veterinary Science, University of Pretoria, Private Bag X04, Onderstepoort, 0110 South Africa; 2 EcoHealth Alliance, 460 West 34th Street - 17th Floor, New York, NY 10001 USA; 3 Centre for Emerging Zoonotic & Parasitic Diseases, National Institute for Communicable Diseases, National Health Laboratory Service, Sandringham, Johannesburg South Africa; 4 ExecuVet, Kenilworth Road, Bloemfontein, South Africa

**Keywords:** Viral epidemiology, Viral infection, Risk factors

## Abstract

Rift Valley fever (RVF) is a mosquito-borne viral zoonosis showing complex epidemiological patterns that are poorly understood in South Africa. Large outbreaks occur in the central interior at long, irregular intervals, most recently in 2010–2011; however, the level of herd immunity of ruminant livestock, a key determinant of outbreaks, is unknown. During 2015–2016 a cross-sectional study on 234 randomly-selected farms investigated the prevalence, patterns of, and factors associated with, antibodies to RVF virus (RVFV) in livestock in an area heavily affected by that outbreak. A RVFV inhibition ELISA was used to screen 977 cattle, 1,549 sheep and 523 goats and information on potential risk factors was collected using a comprehensive questionnaire. The estimated RVFV seroprevalence, adjusted for survey design, was 42.9% in cattle, 28.0% in sheep and 9.3% in goats, showing a high degree of farm-level clustering. Seroprevalence increased with age and was higher on private vs. communal land, on farms with seasonal pans (temporary, shallow wetlands) and perennial rivers and in recently vaccinated animals. Seropositivity amongst unvaccinated animals born after the last outbreak indicates likely viral circulation during the post-epidemic period. The current level of herd immunity in livestock may be insufficient to prevent another large outbreak, should suitable conditions recur.

## Introduction

Rift Valley fever (RVF) is an arthropod-borne viral zoonosis caused by RVF virus (RVFV), a member of the *Phlebovirus* genus, family *Phenuiviridae* of the recently established order *Bunyavirales*^1^. RVF is endemic primarily in sub-Saharan Africa but has crossed several barriers including the Sahara to Egypt, the Red Sea to the Arabian Peninsula and the Indian Ocean to the Comoros, Mayotte and Madagascar^2–4^. Spread through infected animals and mosquitoes to countries where the disease is not endemic is increasingly possible, with more frequent export of livestock from Africa to other countries and the presence of known or potentially competent vectors in those countries^4,5^. RVFV causes sporadic outbreaks with high morbidity and mortality, characterized by abortion storms and high mortalities in neonatal sheep, cattle and goats^6,7^. Human infection generally occurs concurrently with disease outbreaks in domestic ruminants^2,5,8^, where humans work or live in close contact with livestock.

Outbreaks tend to occur following above average rainfall and localized flooding^9^. These climatic conditions favour breeding of floodwater mosquitoes that are the proposed maintenance vectors of this virus via transovarial transmission^10^. The floodwater mosquitoes considered to be vectors on the interior plateau of South Africa are those in the *Aedes* subgenera *Ochlerotatus* and *Neomelaniconion*^11^. Outbreaks may then be amplified by epidemic vectors, of which *Culex theileri* is considered the most important on the interior plateau^12^. Risk factor studies conducted during and after outbreaks in both humans and animals have identified several other environmental, human, and animal factors that may be associated with RVF outbreaks and RVFV seropositivity^13–17^. The presence of large water bodies was found to be associated with seropositivity in Somalia^15^ and southwest Saudi Arabia^16^. Other environmental factors include vegetation density, topography, land use, drainage^14^, temperature^17^ and mosquito abundance^16^. Animal factors that may play a role are animal density^16^, vaccination status^6^ and age^13,18–22^.

RVF was first reported in South Africa (SA) when a large outbreak occurred in 1950–1951^23^. Since then there have been two further large outbreaks, in 1973–1976 and 2008–2011^24^ with the last major outbreak waves occurring in 2010–2011. All three major outbreaks in SA have involved mainly the temperate central interior of SA, centred on the northern and western Free State and adjacent regions of the Northern and Eastern Cape^24^. During the most recent outbreak period (2008–2011), 302 human cases and 25 human deaths (8%) were reported^25^ and in 2010 alone, 14,342 animal cases (of which 13,117 were sheep) and 8,877 (62%) animal deaths were officially reported^24^.

In contrast with East Africa, where interepidemic periods range between 1 and 7 years^26^, large outbreaks in the central interior of SA have occurred only every 20–30 years. The cold, dry climate during the winter is less favourable for survival of mosquito vectors; it is therefore unclear whether, how and for how long the virus survives in the interepidemic periods and whether and to what extent interepidemic circulation of virus occurs^2,6^. Studies elsewhere in Africa have reported varying RVFV seroprevalences in livestock during interepidemic periods. Most studies including multiple species have reported higher seroprevalences in sheep than cattle^15,27,28^, except in Burkina Faso^19^ and south-western Uganda^13^ where there was a higher prevalence in cattle. In an area of Mozambique, a higher seroprevalence was reported in sheep than in goats in 2007^20^ and in 2013^18^, but *vice-versa* in 2010^20^. Despite the fact that the herd immunity of domestic ruminant populations is a key determinant of outbreak occurrence and extent^17^ there are no published studies on the seroprevalence of antibodies to RVFV in the central interior of SA. Although effective vaccines are available, they are generally infrequently used in SA except in the face of an outbreak^6^.

Since the last major outbreak ended in 2011, no further cases of RVF have been reported in SA, except for an outbreak on a single sheep farm in the western Free State in April 2018, which resulted in a limited number of RVFV infections in humans^29^. The level of herd immunity amongst domestic ruminants in outbreak-prone areas of the country is unknown. Therefore, the objective of this study was to estimate the prevalence of antibodies to RVFV in domestic cattle, sheep and goats in a study area in the outbreak-prone central interior of SA, and to identify factors associated with seropositivity.

## Results

### Seroprevalence and clustering

**Figure 1:**
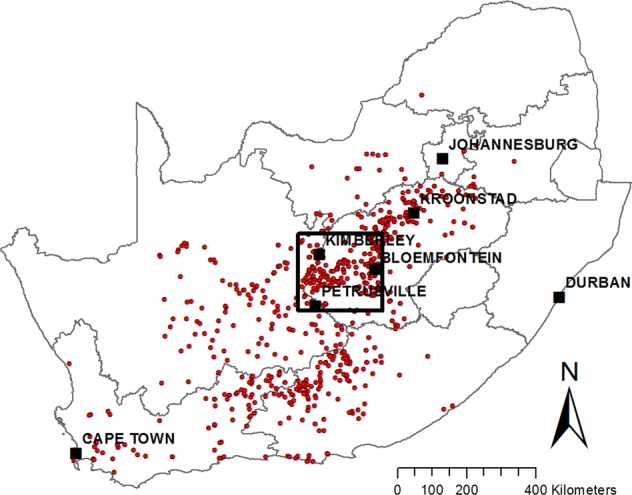
Study area (black rectangle) showing major towns and locations of the RVF outbreaks reported to the OIE during 2010–2011 (red points). The map was constructed for this publication in Esri ArcGIS 10.2 (https://www.esri.com) using country and province boundaries from Esri ArcGIS Online (https://www.esri.com/en-us/store/arcgis-online). Locations of 2010–2011 RVF outbreaks were obtained from http://www.oie.int/wahis_2/public/wahid.php/.

Within our study area (Fig. 1) 3,049 animals (977 cattle, 1,549 sheep, and 523 goats) were sampled from 234 farms and tested for antibodies against RVFV using an inhibition ELISA. The crude proportion of positive samples was 20.4% (622/3049), while 31.8% (311/977) cattle, 16.5% (255/1549) sheep, and 10.7% (56/523) goats were positive.

After adjustment for survey design, including sampling fraction and clustering within herd, overall RVFV seroprevalence in domestic ruminants was estimated to be 29.7% (95% CI: 23.9–36.0%). It was 42.9% (95% CI: 35.7–50.4%) in cattle, 28.0% (95% CI: 21.3–35.4%) in sheep, and 9.3% (95% CI: 5.8–13.9%) in goats. Estimates of herd-level intra-cluster correlation coefficient (ICC, *ρ*) on the prevalence scale, including only herds or flocks that were not allowed to mix with other species, were 0.26 for cattle (74 herds), 0.19 for sheep (66 flocks) and 0.29 for goats (27 flocks). On the 155 farms on which vaccination was reported never to have occurred, the estimates of *ρ* on the prevalence scale for the three species were 0.26, 0.16 and 0.11, respectively. Estimates of *ρ* on the logistic scale (*ρ*_(*l*)_), for unvaccinated, single-species herds or flocks, were 0.36 (95% CI: 0.22–0.54) for cattle, 0.41 (95% CI: 0.25–0.59) for sheep and 0.22 (95% CI: 0.05–0.61) for goats.

**Figure 2:**
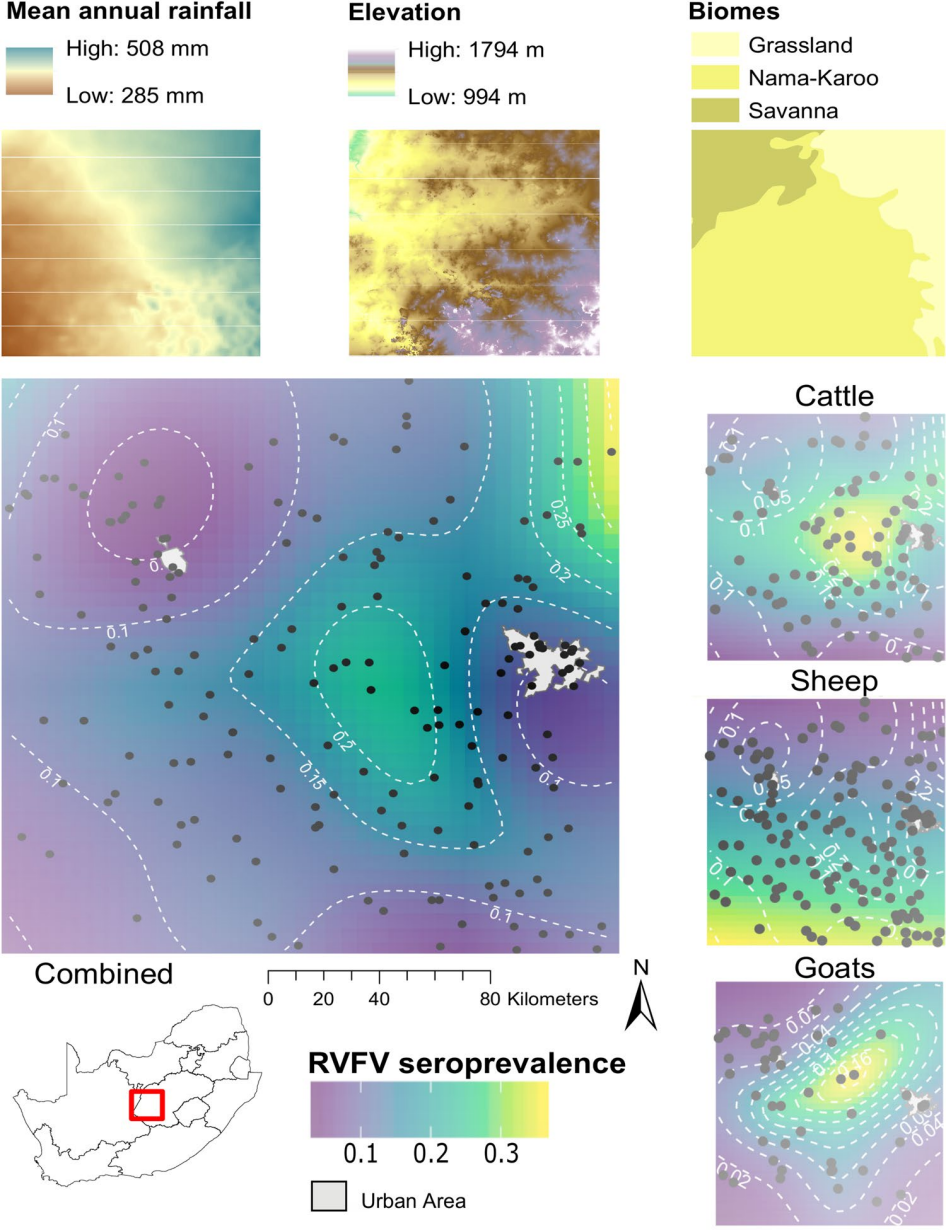
Geographic distribution of RVFV seropositivity across the study area in all unvaccinated livestock, and in cattle, sheep and goats, produced using a generalized additive model with a Gaussian process basis function. Black dots indicate sampling locations; grey areas demarcate major urban and suburban areas (Bloemfontein and Kimberley). Top panels show mean annual rainfall, elevation above mean sea level and major biomes for the study area. The map was constructed for this publication in Esri ArcGIS 10.2 (https://www.esri.com) and R version 3.5.1^66^, using the packages “ggplot2”, “gridExtra”, “rgdal”, “sp”, “raster”, “metR”, “ggspatial” and “viridis” (https://cran.r-project.org/web/packages/) and assembled in Inkscape 0.92 (https://inkscape.org), using country and province boundaries, rainfall and elevation data from Esri ArcGIS Online (https://www.esri.com/en-us/store/arcgis-online), biome and urban area data (https://africaopendata.org) available under a Creative Commons Attribution (CC BY 4.0) license, and coordinates recorded on the farms during the study.

The raster of predicted RVFV seroprevalence produced by spatially-explicit generalized additive models (Fig. 2) showed a general increase in seroprevalence from south-west to north-east across the study area. The patterns differed somewhat between the species, with higher seroprevalence in cattle in the north-east, in sheep in the south and in goats in the central part of the study area.

No apparent clinical signs of RVF were observed on the farms during the survey. Seroprevalence on farms on which RVF had previously been confirmed was 24.7% (95% CI: 21.9–27.7%) compared to 20.9% (95% CI: 19.1–22.9%) on farms where it was not known to have occurred (*P* = 0.027) (Table 1). Seroprevalence on farms that reported abortions during the past three months was 24.6% (95% CI: 21.6–27.9%) vs. 19.1% (95% CI: 17.5–20.8%) on farms without abortions (*P* = 0.001) (Table 1). Amongst animals that (1) were <4 years old, i.e. born since the last outbreak, (2) were reported never to have been vaccinated against RVFV, (3) were born on the farm, and (4) were on farms that reported not vaccinating against RVFV after 2011, 54/862 (6.3%; 95% CI: 4.7–8.1%) were seropositive, with animals 2–4 y old (9.4%; 95% CI: 6.6–12.8%) more likely to be seropositive than those <2 y old (3.9%; 95% CI: 2.4–6.0%) (Fisher’s exact *P* = 0.002).

**Table 1:** Univariate associations of potential risk factors with seropositivity to Rift Valley fever virus in cattle, sheep and goats in central South Africa, 2015–2016.

Variable and level	Number of animals sampled	Number testing positive	% seropositive	*P*-value^†^
District*				<0.001
(19 districts)	3,049	622	20.4	
Year sampled*				0.019
2015	2773	550	19.8	
2016	276	72	26.1	
Species*				<0.001
Cattle	977	311	31.8	
Sheep	1549	255	16.5	
Goats	523	56	10.7	
Breed*				<0.001
Indigenous	1132	153	13.5	
Exotic	1043	258	24.7	
Cross	585	163	27.9	
Age*				<0.001
<2y	929	40	4.3	
2–4y	811	97	12.0	
>4y	1294	484	37.4	
Sex*				<0.001
Female	2704	590	21.8	
Male	343	32	9.3	
Animal born on farm*				0.142
No	464	104	22.4	
Yes	2262	438	19.4	
Animal vaccinated against RVF*				<0.001
No	1892	278	14.7	
Yes	626	226	36.1	
Unknown	531	118	22.2	
Type of farm*				0.001
Communal	308	17	5.5	
Private	2729	604	22.1	
Production system*				<0.001
Commercial	1790	436	24.4	
Semi-commercial	733	115	15.7	
Feedlot	202	53	26.2	
Communal	308	17	5.5	
Main industry*				0.001
Meat	1711	361	21.1	
Wool	663	147	22.2	
Dairy	62	28	45.2	
Other	289	68	23.5	
Animals mix with other domestic ruminants				0.279
No	1647	349	21.2	
Yes	1389	274	19.6	
Animals mix with wildlife*				0.001
No	1592	290	18.2	
Yes	1444	331	22.9	
Animals kraaled at night*				<0.001
No	2232	511	22.9	
Yes	751	97	12.9	
Contact with animals on another farm				0.699
No	2402	495	20.6	
Yes	635	126	19.8	
Animals have access to perennial spring, lake or pond				0.263
No	1836	363	19.8	
Yes	1150	247	21.5	
Animals have access to perennial river or stream*				0.011
No	1992	380	19.1	
Yes	994	230	23.1	
Animals have access to seasonal pan*				<0.001
No	1923	338	17.6	
Yes	1063	272	25.6	
Animals have access to manmade water source (dam)				0.405
No	761	147	19.3	
Yes	2225	463	20.8	
New cattle brought onto farm in past 12 months*				<0.001
No	1956	362	18.5	
Yes	1074	259	24.1	
New goats brought onto farm in past 12 months*				0.040
No	2688	565	21.0	
Yes	345	56	16.2	
New sheep brought onto farm in past 12 months				0.272
No	2026	403	19.9	
Yes	1007	218	21.6	
Quarantine practiced when introducing animals*				0.014
No	2276	442	19.4	
Yes	757	179	23.6	
Vehicles cleaned and disinfected before and after transporting animals*				<0.001
No	1637	290	17.7	
Yes	1396	331	23.7	
Mosquito or fly control practised*				0.103
No	1353	259	19.1	
Yes	1680	362	21.5	
Animals slaughtered on farm*				0.071
No	825	201	24.4	
Yes	1900	403	21.2	
Year of last RVF vaccination on farm*				0.003
Never	2018	387	19.2	
2009–2011	442	90	20.4	
2012–2013	263	51	19.4	
2014–2016	308	90	29.2	
Unknown	18	4	22.2	
Farm size				0.331
<400 ha	565	108	19.1	
400–1000 ha	665	133	20.0	
1001–3000 ha	1021	232	22.7	
>3000 ha	714	148	20.7	
Total number of animals on farm*				0.001
1–100	689	103	14.9	
101–300	690	145	21.0	
301–1000	741	165	22.3	
>1000	929	209	22.5	
Any animals aborted in past 3 months**				0.001
No	2290	438	19.1	
Yes	743	183	24.6	
RVF ever confirmed on farm**				0.027
No	1844	386	20.9	
Yes	881	218	24.7	

^†^*P*-value for Fisher’s exact test.

*Variable selected for inclusion into multiple logistic regression model (*P* < 0.20).

**Variable considered a consequence rather than a potential risk factor, therefore not considered for multivariable model.

Of all animals reported to have been vaccinated against RVFV, 36.1% (95% CI: 32.3–40.0%) were seropositive, compared to 14.7% (95% CI: 13.1–16.4%) of those reported as unvaccinated (*P* < 0.001) (Table 1). Excluding animals with unknown vaccination status, approximately 28% of cattle, 27% of sheep and 11% of goats were reported to have ever been vaccinated against RVFV.

### Risk factor analysis

Based on univariate associations with RVFV seropositivity (Table 1), several variables were selected for inclusion in the multiple logistic regression model. The final multilevel logistic regression model (Table 2) identified significant (*P* < 0.05) associations of six variables with odds of RVFV seropositivity. After adjustment for the other variables in the model, cattle were more likely to be seropositive than both sheep and goats (*P* < 0.001), with the difference between sheep and goats not statistically significant (*P* = 0.142). Odds of seropositivity increased with age (*P* < 0.001) and was higher on private farms than on communal farms (*P* < 0.001), in animals that had access to seasonal pans (temporary, shallow wetlands) (*P* = 0.006) or perennial rivers (*P* = 0.042), and on farms with a history of vaccination within the past two years (*P* = 0.040). The random effect for farm nested within district was highly significant (*P* < 0.001).

**Table 2:** Final multilevel logistic regression model of factors associated with seropositivity to Rift Valley fever virus in cattle, sheep and goats in central South Africa, 2015–2016, adjusted for clustering within farms.

Variable and level	OR	95% CI	*P*-value
Species			
Cattle	1*		
Sheep	0.48	0.32–0.72	<0.001
Goats	0.30	0.16–0.56	<0.001
Age (years)			
<2	1*		
2–4	2.82	1.79–4.44	<0.001
>4	17.08	11.29–25.85	<0.001
Type of farm			
Communal	1*		
Private	4.78	2.02–11.29	<0.001
Access to perennial river			
No	1*		
Yes	1.53	1.02–2.31	0.042
Access to seasonal pan			
No	1*		
Yes	1.75	1.17–2.61	0.006
Year of last RVF vaccination on farm			
Never	1*		
2009–2011	0.96	0.56–1.65	0.893
2012–2013	0.90	0.45–1.78	0.760
2014–2016	1.86	1.03–3.36	0.040
Unknown	2.71	0.26–28.89	0.408

Variance of random effect for farm nested within district = 1.48 (95% CI: 1.06–2.07; P < 0.001).

Model log-likelihood = −1,114.935; AIC = 2,255.87; *n* = 2,971.

*Reference category.

OR = odds ratio, CI = confidence interval, AIC = Akaike’s information criterion.

In a separate multilevel logistic regression model including only unvaccinated animals <4 years old, born on farms that had not vaccinated against RVFV since 2011 (Table 3), the odds of seropositivity was higher in cattle than in sheep (*P* = 0.032) and in animals 2 y or older (*P* = 0.031). In addition, animals with access to a perennial river on the farm were more likely to be seropositive (*P* = 0.044).

**Table 3:** Factors associated with seropositivity to Rift Valley fever virus in unvaccinated domestic ruminants born after the last outbreak in central South Africa, adjusted for clustering within farms.

Variable and level	OR	95% CI	*P*-value
Species			
Cattle	1*		
Sheep	0.23	0.06–0.88	0.032
Goats	0.78	0.33–1.85	0.572
Age (years)			
<2	1*		
2–4	2.16	1.07–4.34	0.031
Access to perennial river			
No	1*		
Yes	2.76	1.03–7.37	0.044

Variance of random effect for farm nested within district = 2.22 (95% CI: 0.99–5.01; P < 0.001).

Model log-likelihood = −176.992; AIC = 368.956; *n* = 854.

*Reference category.

OR = odds ratio, CI = confidence interval, AIC = Akaike’s information criterion.

## Discussion

This study is the first to estimate RVFV seroprevalence in domestic ruminants in the outbreak-prone central interior of SA and provides an estimate of the seroprevalence during an interepidemic period, four years after the last outbreak, as well as evidence of its association with certain factors. The overall individual animal seroprevalence of RVFV four years after the last outbreak was 29.7%, and it was 43.0% in cattle, 28.0% in sheep, and 9.3% in goats. For cattle and sheep, these results are comparable to seroprevalences reported in 2013 from Kenya^30,31^ and Tanzania^32^, six years after the 2006/2007 East African RVF outbreak, bearing in mind that some of the animals in this study were vaccinated for RVF whereas vaccinated herds were excluded from the studies in Kenya and Tanzania^31,32^. After adjustment for confounding, cattle were more likely to be seropositive than both sheep and goats. The differences in seroprevalence between species may be attributed to several factors, including differential vector preference and differences in management system. Several other studies have also reported lower seroprevalences in goats, in Kenya^33^, Rwanda^34^, Comoros^35^, Mozambique^18^ and Mauritania^36^. In our study, the higher seroprevalence in cattle, and to a lesser extent sheep, may be partially explained by the fact that cattle and sheep are predominantly commercially reared and farmers in general may place more value on them than on goats; therefore, they were more likely to be vaccinated. Although vaccination history was obtained in the survey and included in the final multivariable model, in most cases the accuracy of this information was dependent on the farmers’ recall and misclassification of exposure may have occurred. There is no method for differentiation of vaccinated from infected animals (DIVA), making it impossible to distinguish between antibodies derived from vaccination or natural RVFV infection.

The highest seroprevalence was found in animals older than four years in all species tested. This is to be expected since they survived the last outbreak during which they may have been exposed to RVFV and/or vaccinated. However, the 6.3% seropositivity found in unvaccinated cattle, sheep, and goats born since the last outbreak, indicates that subclinical RVFV circulation might have occurred in the study area. This is supported by the fact that, within this sub-group the seroprevalence increased with age, with animals born shortly after the last outbreak more likely to have been exposed. Unreported or subclinical cases are therefore likely to have occurred after the last outbreak. In addition, the association of seropositivity with the recent occurrence of abortions on the farm indicated the possibility of sporadic, unreported cases of RVF having occurred up to four years after the outbreak.

In the absence of a chronically infected vertebrate host, survival of the virus through the dry winter period immediately after the outbreak season is believed to be facilitated by overwintering vectors^6^. Two hypotheses have been proposed for RVFV, with varying degrees of investigative evidence, namely survival of the virus in embryonated eggs laid in the dry margins of seasonal wetlands (infected transovarially)^10^ and survival of virus in overwintering *Culex* mosquitoes^37^. The reproductive biology of the members of the *Aedes* genus, requiring drying of eggs prior to hatching, favours the former hypothesis and that of the multivoltine genus, *Culex*, favours the latter^38^. Evidence for overwintering of RVFV in adult mosquitoes is lacking but the flavivirus, West Nile virus, has been proven experimentally and observationally to overwinter in *Culex* species in the temperate regions of the northern hemisphere^39,40^. In South Africa, quiescent adult female *Culex* spp., including *Cx. theileri*, considered to be the main epidemic vector on the inland plateau^12^, have been shown to overwinter^41,42^. Typical hibernacula for adult mosquitoes include caves, tree-holes and building infrastructure^43^. In the drier interior of South Africa such refuges are restricted to farmsteads or livestock enclosures, although trees do cluster along river banks, which may help explain the association between access to perennial rivers and seropositivity in unvaccinated animals born after the last outbreak.

Domestic ruminants pastured on private grazing land showed higher seropositivity compared to those reared on communal land. Communal farmers, who were likely less informed about the economic importance of RVF, were less likely to have vaccinated their animals. Although vaccination history was included in the multivariable model, it is likely that inaccuracy in these data, as well as the fact that the majority of communal farmers were unsure of their animals’ vaccination status, resulted in failure to fully account for this confounding. It is also likely that private farms were in general situated in better farming areas, with more water available and therefore more suitable for vector breeding and disease transmission. As expected, higher odds of seropositivity were found in domestic ruminants that had access to seasonal pans and rivers on the farm, where mosquito vector abundance is likely to be higher and which is a putative RVF risk factor^6,14,16,44^.

Vaccination was associated with RVFV seropositivity only if it had been done within the past 1–2 years, and only 37% of animals reported to have been vaccinated against RVFV were seropositive. Although, as discussed above, vaccination history may sometimes not have been reliable, this raises questions regarding the efficacy of vaccines administered on farms, which may have been affected by conditions during transport, storage, handling and administration. The live Smithburn RVF vaccine has been reported to evoke long lasting, and even lifelong, immunity, but with a poorer antibody response in cattle^6^. Seropositivity after vaccination with Clone 13 RVF vaccine has been reported to persist for at least 1 year, but the vaccine induces different levels of immune responses in domestic ruminants, being highly immunogenic in sheep and goats and moderately so in cattle^45,46^. Formalin-inactivated RVF vaccine has been reported to induce high antibody titres and evoke protection for 9 months in cattle, and up to 2 years if a booster is given after 3 months^47,48^. Further investigation of the duration of immunity following administration of different vaccines is required.

The sampling during this study extended from mid-October 2015 to late February 2016. The difference on univariate analysis between seroprevalence in animals sampled in 2015 (19.8%) vs. 2016 (26.1%) raises the possibility that seroprevalence increased over the course of the study, either due to vaccination or natural exposure. However, year was non-significant when included in the multivariable model (*P* = 0.825), indicating that this was more likely due to confounding with spatial or other factors. In addition, rainfall over the entire study area during the sampling period was well below average^49^, suggesting that mosquito vector abundance would also have been lower than usual and therefore natural viral circulation less likely.

The difference in seroprevalence by sex in the univariate analysis was no longer evident when included in the multivariable model and was therefore likely due to confounding by other variables. Some other studies have reported higher seroprevalence in females than in males^20,21,30^, but the reasons for this are unclear and the apparent differences in exposure are likely due to differences in management.

The general pattern of increasing seroprevalence from south-west to north-east across the study area is consistent with the geographic trend in rainfall, which likely correlates with the occurrence and extent of suitable habitat for mosquito vectors. However, the variation in RVFV seroprevalence between farms, with estimates of ICC on the prevalence scale for unvaccinated animals between 0.11 and 0.26, indicate that, even within an area prone to RVF outbreaks, the occurrence of infection varies greatly between locations, as reported elsewhere^15,32,50^. Care should be taken when comparing published ICCs since some authors report the statistic on the logistic scale, calculated during the estimation of a multilevel logistic regression model. For example, Bett *et al*.^30^ in Kenya reported herd- and village-level ICCs on the logistic scale (*ρ*_(*l*)_) of 0.3 and 0.22 respectively; our estimates of *ρ*_(*l*)_, ranging between 0.22 and 0.41, were somewhat higher, reflecting greater between-herd variation in seroprevalence than in Kenya, where outbreaks occur more frequently and conditions are likely more conducive to interepidemic viral circulation and widespread exposure^26^. Vaccination may also artificially inflate estimates of *ρ*, evident in the reduction in estimated *ρ* when vaccinated herds/flocks were excluded, as when farmers vaccinate they are likely to vaccinate all of the animals at risk, and even if they vaccinate a portion every year it is likely that the older animals were vaccinated previously. In our study area, the presence of large areas without surface water is likely to have resulted in spatial variation in distribution of mosquito vectors. Further, fine-scale differences in local ecological conditions, agricultural practices and type of surface water would have influenced vector abundance and resulted in marked spatial heterogeneity of RVFV exposure.

The complexity of the above factors makes it difficult to predict the location and timing of outbreaks. This is illustrated by the recent occurrence of a small outbreak within the study area in April 2018 at the end of the rainy season, affecting only one farm^51^. Attempts to improve our understanding of factors responsible for initiation of outbreaks will likely need to include detailed studies of the ecology and distribution of vectors, their habitats and determinants of their abundance. Further, identifying the geographic determinants of outbreaks will require spatial models with geographic explanatory variables linking these detailed studies at the regional level.

On a broader scale, the herd immunity threshold (HIT) is defined as the proportion of the host population required to be immune in order to control transmission and prevent an outbreak^52^. It depends on *R*_0_, the basic reproduction number, which is defined as the expected number of hosts infected by a single infected host in a fully susceptible population^53^. For a vector-borne disease, *R*_0_ is determined by several factors which may vary in different settings, including the vector to host ratio, the biting rate and the vector survival rate^54^. The HIT for RVF near the start of the 2010 outbreak in our study area was previously estimated to be between 50% and 85%, based on an estimated effective reproduction number that peaked at 4.3 in February 2010^17^. Estimated seroprevalence in our study was well below this range in all three species, indicating that, should conditions similar to those in early 2010 recur, the likelihood of another large outbreak may be high.

## Conclusions

The overall prevalence of antibodies to RVFV amongst domestic livestock in the central interior of SA four years after the end of a major outbreak was found to be 29.7% and was highest in cattle and lowest in goats. The presence of RVFV IgG antibodies in domestic ruminants born after the last outbreak, and the association of seropositivity with known environmental risk factors for RVF transmission indicate the possibility that viral circulation has taken place during the inter-epidemic period. The current seroprevalence may be below the herd immunity threshold required to prevent another large outbreak should suitable conditions recur and is likely to decline further unless effective vaccination of susceptible livestock is undertaken on a large scale.

## Methods

### Study setting and ethical approvals

This was part of an integrated, multidisciplinary One Health study entitled “Understanding Rift Valley Fever in the Republic of SA”. The project protocol adhered to the specifications of the South African National Standard (SANS 10386-2008): “The Care and Use of Animals for Scientific Purposes”, and approval was obtained from the University of Pretoria Animal Ethics Committee (t005-16, V013-15, V090-15), Tufts University Institutional Animal Care and Use Committee (G2014-03 and G2016-148), and the US Army Medical Research and Materiel Command Animal Care and Use Review Office (CT-2014-33).

### Study area

A cross-sectional study was conducted during 2015–2016 within a ~40,000 km^2^ study area between Bloemfontein and Kimberley in the central interior of SA, an area heavily affected by the 2010–2011 RVF outbreaks (Fig. 1). Bloemfontein is the capital city of Free State Province, situated on dry savannah at 29.1°S, 26.2°E, at an altitude of 1,395 m above sea level. Kimberley is the capital of Northern Cape Province, located at 28.75°S, 24.75°E, approximately 110 km east of the confluence of the Vaal and Orange Rivers at an altitude of 1,224 m above sea level. The study area lies at the intersection of the Nama-Karoo biome in the centre and south, the Savanna biome in the north-west and the Grassland biome in the east. The climate is semi-arid, with mean annual rainfall varying between 285 mm in the south-western corner to 500 mm in the north-east. The area is traversed by several perennial rivers, with the two largest rivers in SA, the Orange and Vaal, crossing the south-western and north-western corners of the study area, respectively. The landscape is also characterized by the presence of numerous temporary, shallow wetlands, or “pans”, of various types, most of which contain water only during times of unusually high rainfall^55^.

### Study design and sampling strategy

The target population was all cattle, sheep and goats in the study area, including those on commercial, smallholder and communal farms. A two-stage random sampling strategy was used. Random geographic points were used to select farms since no sampling frame of all farms in the study area existed. Random points were generated within the study area, with selection probability proportional to the density of livestock-owning households obtained from the results of the 2011 National Census (K. Parry, Statistics South Africa, 2014, *pers. comm*.). This was done by first excluding census units (known as small areas) less than ~1 km^2^ in extent which, based on Google Earth imagery (http://earth.google.com), represented urban areas, mainly in Bloemfontein and Kimberley. For each of the remaining 262 small areas, a Poisson-distributed random number with mean proportional to the number of livestock-owning households was generated in order to determine the number of points to be generated in each. 350 random geographic points were thus generated using Arc Toolbox in ArcGIS 10.2 (ESRI, Redlands, CA, U.S.A.). Since the exact number of farms to be sampled in order to achieve the required number of each species was unknown, the list was randomly sorted and the points were then sampled in the sequence they appeared on the list; this ensured that the points actually used constituted a random sample. Each point was plotted on a Google Earth map of the study area and the farm nearest to each selected point was identified with the help of state veterinary officials.

### Sample size

Sample size to estimate a proportion with 95% confidence was calculated for each species as follows^56^:

$$n=\frac{{1.96}^{2}\times {P}_{exp}(1-{P}_{exp})}{{d}^{2}}$$

where *n* = required sample size, *P*_*exp*_ = expected prevalence and *d* = desired absolute precision. Sample size was multiplied by the design effect (*D*) for multi-stage-stage sampling, calculated as follows^57^:

$$D=1+\rho (n-1)$$

where *ρ* is the intra-cluster correlation coefficient (ICC) and *n* is the average cluster size. Due to lack of recent data on seroprevalence of RVF in cattle, sheep, and goats in southern Africa, *P*_*exp*_ of 15% for cattle and goats and 25% for sheep were used, based on previously reported seroprevalence elsewhere in Africa. Desired precision was 5%. The ICC for RVF is unknown but for most diseases is unlikely to exceed 0.2^58^; therefore, using *ρ* = 0.2 and *n* = 9, *D* was calculated as 2.6, and minimum required sample size was 510 for cattle and goats, and 752 for sheep.

### Animal sampling

Animals were sampled on each selected farm, stratified by age category: 6 months to 2 years (in order to exclude animals with maternal antibodies), 2–4 years (adults born since the last outbreak) and >4 years (alive during the last outbreak in 2011). Where possible, systematic random sampling was done, with three animals of each species selected within each age category. In many cases, however, a combination of haphazard and convenience sampling was used, with farm workers selecting animals from each age category. If the required number of animals in each age group was not available, additional animals were selected from the other age groups. With some exceptions, explained below, a maximum of nine cattle, nine sheep and, nine goats were sampled per farm. In cases where the number of cattle, sheep or goats on the selected farms was less than nine, all the available cattle, sheep or goats were sampled. On farms that reported that they had never vaccinated their animals, up to 50 sheep were sampled, if available, in order to identify seronegative sheep for a parallel cohort study that is still ongoing and will be reported separately. Sampling of farms continued, using the randomly sorted list of co-ordinates, until the minimum required number of each species was achieved. Because goats were the least frequently encountered species, this resulted in greater than the required number of cattle and sheep being sampled. More than nine goats were sampled on some farms in order to achieve the required sample size.

Blood samples were collected by venipuncture from the jugular or caudal vein into sterile 8.5 mL evacuated tubes (Vacutainer, BD) with clot activator and gel for serum separation and allowed a minimum of 15 minutes to clot. The samples were then centrifuged using a portable centrifuge (Beckman Coulter Allegra X-22) for 15 min at 1200 g and packaged in a cooler box with ice packs for transportation to the National Institute for Communicable Diseases Special Viral Pathogens Unit, Johannesburg. The serum was then aliquotted and stored at −20 °C until analysed.

### Questionnaire

A questionnaire to collect information concerning animal, management, and environmental factors was designed with closed-ended questions and translated into local languages (seSotho, English, and Afrikaans). The questionnaire was piloted on 14 farms just outside the study area. It was administered to farm owners or managers in an interactive format using an electronic tablet by trained individuals and the responses were uploaded into the project database via the internet for storage. The wording of the same questions was modified slightly between questionnaires for domestic farms on private land and those that used communal land. A copy of the questionnaire is given in the Supplementary Information.

### Laboratory analysis

An inhibition enzyme-linked immunosorbent assay (iELISA) based on tissue culture-derived whole virus RVFV antigen, with reported diagnostic sensitivity of 93–100% and diagnostic specificity of 99–100%^59,60^, was carried out as previously described with minor modification^59^. Briefly, 96-well ELISA plates (Nunc MaxiSorp, Nunc, Denmark) were coated overnight at 4 °C with 100 μL per well of polyclonal sheep anti-RVFV capture antibody diluted 1:500 in phosphate buffered saline (PBS without Ca and Mg, pH 7.4). Plates were washed three times with 300 μL per well of wash buffer (0.1% Tween-20 in PBS) after overnight incubation. Plates were blocked with 10% skimmed milk in PBS (blocking buffer) and incubated for 1 hour at 37 °C in a humidified chamber, followed by washing as described above. Test and control serum dilutions were prepared by adding 21 μL of undiluted serum into 189 μL of virus or mock antigen diluted 1:10 in diluent buffer (2% skimmed milk in PBS). These sample-antigen dilutions were added in duplicate (100 μL per well) to the washed plate after blocking and incubated for 1 hour at 37 °C in a humidified chamber, followed by washing as described above. Rabbit polyclonal antiserum to the RVFV nucleoprotein at a dilution of 1:2000 was added to all the wells (100 μL per well) and incubated for 1 hour at 37 °C in a humidified chamber, followed by washing as described above. Horseradish peroxidase conjugated anti-rabbit antibody (KPL) diluted 1:6000 was added to all the wells (100 μL per well) and incubated for 1 hour at 37 °C in a humidified chamber, followed by washing as described above. Peroxidase substrate (ABTS, KPL) was added to each well (100 μL) and plates incubated in the dark at room temperature (22–24 °C) for 30 minutes, followed by addition of stop solution (1% sodium dodecyl sulphate). Optical density was measured at 405 nm and the specific activity of each serum calculated by subtracting the OD in the mock antigen well from that in the RVFV antigen well. The mean net OD readings for replicate tests were converted to percentage inhibition value using the equation [(100 − (mean net OD of test sample/mean net OD of negative control)) × 100]. Samples were categorized as positive or negative based on species-specific cut-off values established previously^59^.

### Data analysis

Questionnaire data were collected using an Android tablet running Open Data Kit software^61^. Data were downloaded, cleaned and combined with the laboratory results using RStudio^62^, and exported to a CSV file and Microsoft Excel was used for additional cleaning before being transferred into Stata 15 (StataCorp, College Station, TX, U.S.A.) for analysis.

Analyses were performed for cattle, sheep and goats combined. An animal was defined as seropositive to RVFV if it tested positive using the iELISA. Sampling fraction for each herd was calculated as the proportion of animals sampled in the herd, where a herd was defined as all the animals of one species present on a farm. Overall and species-specific seroprevalences were adjusted to remove the potential bias due to unequal sampling fractions by weighting each observation by the sampling weight, calculated as the inverse of the sampling fraction. In addition, the standard errors of the estimates were adjusted by using a robust variance estimator to account for the clustered sampling design to produce 95% confidence intervals. These analyses were done using the “*svy*” command in Stata 15.

To estimate the degree of clustering on the prevalence scale, ICC (*ρ*) was calculated separately for each species as follows^63^:

$$\rho =\frac{\mathop{\sum }\limits_{i=1}^{K}\{{Y}_{i+}({Y}_{i+}-1)-2P({n}_{i}-1){Y}_{i+}+{n}_{i}({n}_{i}-1){P}^{2}\}}{\mathop{\sum }\limits_{i=1}^{K}\{{n}_{i}({n}_{i}-1)P(1-P)\}}$$

where *K* is the number of herds/flocks, *Y*_*i*+_ is the number of seropositive animals in herd *i*, *n*_*i*_ is the number of animals tested in herd *i* and *P* is the overall (unadjusted) seroprevalence. The ICC on the logistic scale (*ρ*_(*l*)_), with 95% confidence interval, was calculated separately for each species using the variance of the random effect for herd (*σ*^2^) estimated in an intercept-only, two-level logistic model of the inhibition ELISA result, as follows^64^:

$${\rho }_{(l)}={\sigma }^{2}/({\sigma }^{2}+{\sigma }_{SL}^{2})$$

where *σ*^2^_SL_ = *π*^2^/3, the variance of the standard logistic distribution. This was done using the “*estat icc*” post-estimation command in Stata 15.

On the basis of a putative causal diagram, factors conceivably on, or associated with, the causal pathway for RVFV exposure, as well as factors likely to be consequences of RVFV exposure, were included in a univariate analysis using contingency tables. However, only the former were considered for inclusion into the multiple logistic regression model. Fisher’s exact *P* < 0.2 was used as inclusion criterion in the model. Multivariable analysis was undertaken using multilevel logistic regression models with a random effect for farm nested within district. Manual backward stepwise elimination was performed, with Wald *P* < 0.05 as the criterion for retention in the model. Finally, each eliminated variable was re-tested individually in the model and retained if Wald *P* < 0.05.

To examine and visualize geospatial patterns in seroprevalence amongst unvaccinated livestock, we fit generalized additive models (GAM) with a two-dimensional Gaussian process basis function^65^ to animal-level serological status at farm geographic coordinates, modelling each species (cattle, sheep, and goats) separately as well as all livestock. This analysis was performed in R version 3.5.1^66^ using the “*mgcv*” package (https://cran.r-project.org/web/packages/mgcv). The model was then used to create a raster of predicted seroprevalence over the entire study area for all livestock, and for cattle, sheep, and goats using the R packages “*ggplot2*”, “*gridExtra*”, “*rgdal*”, “*sp*”, “*raster*”, “*metR*”, “*ggspatial*” and “*viridis*” (https://cran.r-project.org/web/packages/).

## Supplementary information

Supplementary information.

## Data Availability

The dataset analysed during the current study is available from the corresponding author on reasonable request.
