# Supplementary material for: Patterns of Rift Valley fever virus seropositivity in domestic ruminants in central South Africa four years after a large outbreak
Source: Sci Rep. 2020 Mar 26;10:5489. doi: 10.1038/s41598-020-62453-6 (PMC7099094; doi:10.1038/s41598-020-62453-6)
Supplement: Supplementary file 1 — Supplementary information. [file 41598_2020_62453_MOESM1_ESM.docx]

Supplementary material

Patterns of Rift Valley fever virus seropositivity in domestic ruminants in central South Africa four years after a large outbreak

Yusuf B. Ngoshe^1^, Alida Avenant^1^, Melinda K. Rostal^2^, William B. Karesh^2^, Janusz T. Paweska^3^, Whitney Bagge^2^, Petrus Jansen van Vuren^3^, Alan Kemp^3^, Claudia Cordel^4^, Veerle Msimang^1,3^, Peter N. Thompson^1^

^1^ Epidemiology Section, Department of Production Animal Studies, Faculty of Veterinary Science, University of Pretoria, Private Bag X04, Onderstepoort 0110, South Africa

^2^ EcoHealth Alliance, 460 West 34^th^ Street - 17^th^ Floor, New York, NY 10001, U.S.A.

^3^ Centre for Emerging Zoonotic & Parasitic Diseases, National Institute for Communicable Diseases, National Health Laboratory Service, Sandringham, Johannesburg, South Africa

^4^ ExecuVet, Kenilworth Road, Bloemfontein, South Africa


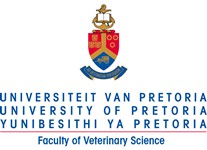


**SEROPREVALENCE AND FACTORS ASSOCIATED WITH SEROPOSITIVITY TO RIFT VALLEY FEVER VIRUS IN DOMESTIC RUMINANTS IN CENTRAL SOUTH AFRICA**

Kindly tick √ □ to indicate that the project details have been explained to you and you consent willingly to participate in this study.

Thank you for your cooperation.

*Date............................*

**SECTION A (OWNER DEMOGRAPHICS)**

1. Name................................................................................
2. Farm name………………………………………………
3. Farm location/district........................................................
4. Total number of animals on farm……………………….
5. Farm size………………………………………………...
6. Type of farm: Communal [ ] Private [ ]
7. Farm GPS coordinates…………………………….........

**SECTION B (HERD DEMOGRAPHICS)**

1. How many of these animals do you have today?

| **Species** | **Cattle** | **Sheep** | **Goat** | **Wild ruminants** | **Others (specify)** |
| --- | --- | --- | --- | --- | --- |
| **Numbers** |  |  |  |  |  |

1. Have there been new animal(s) introduced onto the farm? Yes [ ] No [ ]
2. If yes, please complete:

| **How many animals have been introduced onto the farm?** | | | |
| --- | --- | --- | --- |
| This year | Last year | In the last two years | In the last three years or more |
|  |  |  |  |

**SECTION C (MANAGEMENT SYSTEM)**

1. Type of production system: Commercial [ ] Semi-commercial [ ] Feedlot [ ] Communal [ ] Other (specify)…………
2. Type of farm main industry: Meat [ ] Wool [ ] Dairy [ ] Other (specify)…………………

**SECTION D (ANIMAL MANAGEMENT)**

1. Are your animals born on your farm? Yes [ ] No [ ]
2. How many new cattle brought on to farm in the past 12 months? ………..
3. How many new sheep brought on to farm in the past 12 months? ………..
4. How many new goats brought on to farm in the past 12 months? ………..
5. Do you quarantine new animals before introducing them to your herd? Yes [ ] No [ ]
6. Are your vehicles cleaned before and after transporting animals? Yes [ ] No [ ]
7. Do your animals mix with other domestic ruminants? Yes [ ] No [ ]
8. Do your animals mix with wildlife? Yes [ ] No [ ]
9. Do your animals have access to a perennial spring lake or pan? Yes [ ] No [ ]
10. Do your animals have access to a perennial river or stream? Yes [ ] No [ ]
11. Do your animals have access to a seasonal pan? Yes [ ] No [ ]
12. Do your animals have access to a manmade water source (dam)? Yes [ ] No [ ]
13. Do you slaughter animals on your farm? Yes [ ] No [ ]
14. Do you keep your animals together in the kraal at night? Yes [ ] No [ ]
15. Do your animals have contact with animals on another farm? Yes [ ] No [ ]
16. Do you use mosquito or fly repellent on your animals? Yes [ ] No [ ]
17. Have any of your animals aborted in the past 12 months? Yes [ ] No [ ]
18. Are your animals vaccinated against Rift Valley fever? Yes [ ] No [ ] Unknown [ ]
19. If yes, when was the last year you vaccinated for Rift Valley fever on your farm?............
20. Which vaccine product do you use? Smithburn vaccine [ ] Inactivated vaccine [ ] Clone 13 [ ] Others (specify)…………………
21. Which group of animals were vaccinated? Young cattle [ ] Young goats [ ] Young sheep [ ] New animals [ ] Most valuable animals [ ] Bulls only [ ] Cows only Sheep only [ ] Goats only [ ] Others (specify)…………………
22. Has Rift Valley fever ever been confirmed on your farm? Yes [ ] No [ ]
